# Cancer cell sensitivity to bortezomib is associated with survivin expression and p53 status but not cancer cell types

**DOI:** 10.1186/1756-9966-29-8

**Published:** 2010-01-22

**Authors:** Xiang Ling, Diane Calinski, Asher A Chanan-Khan, Muxiang Zhou, Fengzhi Li

**Affiliations:** 1 Departments of Pharmacology & Therapeutics, Roswell Park Cancer Institute, Buffalo, New York 14263, USA; 2 Department of Medicine, Roswell Park Cancer Institute, Buffalo, New York 14263, USA; 3 The Multiple Myeloma Research Group, Roswell Park Cancer Institute, Buffalo, New York 14263, USA; 4 Department of Pediatrics, Emory University School of Medicine, Atlanta, GA 30322, USA

## Abstract

**Background:**

Survivin is known playing a role in drug resistance. However, its role in bortezomib-mediated inhibition of growth and induction of apoptosis is unclear. There are conflicting reports for the effect of bortezomib on survivin expression, which lacks of a plausible explanation. Methods: In this study, we tested cancer cells with both p53 wild type and mutant/null background for the relationship of bortezomib resistance with survivin expression and p53 status using MTT assay, flow cytometry, DNA fragmentation, caspase activation, western blots and RNAi technology.

**Results:**

We found that cancer cells with wild type p53 show a low level expression of survivin and are sensitive to treatment with bortezomib, while cancer cells with a mutant or null p53 show a high level expression of survivin and are resistant to bortezomib-mediated apoptosis induction. However, silencing of survivin expression utilizing survivin mRNA-specific siRNA/shRNA in p53 mutant or null cells sensitized cancer cells to bortezomib mediated apoptosis induction, suggesting a role for survivin in bortezomib resistance. We further noted that modulation of survivin expression by bortezomib is dependent on p53 status but independent of cancer cell types. In cancer cells with mutated p53 or p53 null, bortezomib appears to induce survivin expression, while in cancer cells with wild type p53, bortezomib downregulates or shows no significant effect on survivin expression, which is dependent on the drug concentration, cell line and exposure time.

**Conclusions:**

Our findings, for the first time, unify the current inconsistent findings for bortezomib treatment and survivin expression, and linked the effect of bortezomib on survivin expression, apoptosis induction and bortezomib resistance in the relationship with p53 status, which is independent of cancer cell types. Further mechanistic studies along with this line may impact the optimal clinical application of bortezomib in solid cancer therapeutics.

## Background

Although bortezomib (PS-341) was largely applied to treatment of hematopoietic malignancy such as myeloma, growing basic studies and clinical trials reveal that bortezomib can be used to treat many types of solid tumors alone and in combination with other chemotherapeutic drugs. This includes colon-gastric cancer [1-3], breast cancer [4-9], prostate cancer [10-14] and lung cancer [15-18] as well as others. Therefore, use of solid tumor-derived cancer cell lines to study the mechanism of bortezomib drug resistance is important for effective application of bortezomib in treatment of patients with solid tumors in the clinic.

Survivin, a unique member of the Inhibitor of Apoptosis (IAP) Protein Family, is cell cycle-regulated [19,20] and its expression in cancer has been associated with cancer progression, drug resistance, and shortened patient survival [21,22]. Given that survivin is highly expressed in malignant cells but is undetectable in most normal adult tissues, it is considered as a potentially important therapeutic target [23]. Survivin antagonizes apoptosis and is involved in the mitotic spindle assembly checkpoint [24,25]. Thus, inhibition of survivin expression or function induces both apoptosis and cell division defect. Many protein factors and signaling transduction pathways can modulate the expression of survivin [26]. For example, it has been reported that p53 transcriptionally downregulates the expression of survivin in various cancer cells with wild type p53 [27-29], and the inhibition of survivin by p53 can be reversed by growth-stimulatory factors such as estrogen receptor-α [30].

While survivin is a known universal drug resistant factor, the role and expression for survivin in bortezomib-induced cancer cell growth inhibition and apoptosis induction remains unclear. Some of the previous reports noted that treatment of cancer cells with bortezomib is associated with enhanced apoptosis and reduced expression of survivin [31,32], while other investigators reported that bortezomib-induced apoptosis is accompanied with an induction of survivin expression in human NSCLC cells [33]. Recently, it has been also reported that the role for survivin in bortezomib-induced apoptosis is cell type-dependent [34]. In this study, we demonstrated that modulation of survivin expression by bortezomib is dependent on p53 status but independent of cancer cell type. In cancer cells with mutated p53 or p53 null, bortezomib appears to induce survivin expression, while in cancer cells with wild type p53, bortezomib either downregulates or shows no significant effect on survivin, which is dependent on cell line, bortezomib concentration and duration of exposure. These findings, for the first time, unified the current different observations about the effect of bortezomib on survivin expression, apoptosis induction and bortezomib resistance, and warranted further mechanistic studies and application of these findings in cancer therapeutics.

## Methods

### Cell culture and reagents

Colon cancer cell lines (HCT116p53^+/+^, HCT116p53^-/-^), lung cancer cell lines (EKVX and A549), prostate cancer cells (PC-3 and LNCaP) and multiple myeloma cell lines (KMS11 and RPMI8226) were maintained in RPMI 1640 medium. Breast cancer cells (MDA-MB-231 and MCF-7) were cultured in DMEM medium. All cell cultural mediums were supplied with 10% fetal bovine serum (FBS, Atlanta Biologicals, Lawrenceville, GA) and penicillin (100 units/ml)/streptomycin (0.1 μg/ml) (Invitrogen, Grand Island, NY). Cells were routinely subcultured twice a week and maintained in a humidified incubator with 5% CO2 at 37°C. Polyclonal anti-actin antibody and goat peroxidase-conjugated anti-rabbit IgG antibody were purchased from Sigma (St. Louis, MO). Survivin antibody (FL-142) was purchased from Santa Cruz (Santa Cruz, CA), MTT (tetrazolium salt, 3- [4,5-dimethylthiazol-2-yl]-2,5,-diphenyltetrazolium bromide) and leupeptin were purchased from Usb (Cleveland, OH).

### Cell treatment and siRNA/shRNA transfection/infection

Cells grown in medium containing 10% serum were treated with and without bortezomib in various concentrations (see text and results) for 24 - 72 hours were harvested and followed by various analyses. siRNA transfection [35] and shRNA infection [36] were performed as previously described

### MTT cell viability assay

Effect of bortezomib on cell growth was determined by MTT assay. MTT was used as a colorimetric substrate for measuring cell viability. Non-viable cells, with altered cellular redox activity, are unable to reduce the MTT dye. After 72 hours with or without bortezomib treatment, MTT was added (to a final concentration of 0.5 mg/ml). Cells in 96-well plates were incubated in a 5% CO_2 _incubator at 37°C for 4 hours, and then lysed thoroughly with lysis buffer (20% SDS, 50% N, N-dimethylformamide, pH 4.7, 100 μl/well). The absorbance in the relevant wells was measured at 570 nm using an Ultra Microplate Reader (Bio-Tek Instruments).

### Flow cytometry analysis

Cells at sub-confluence (~30%) were treated with bortezomib at 0, 5, 10 and 50 nM for 48 hours and then harvested by trypsinization and washed with PBS. Cells (~1 × 10^6^) were resuspended in 5 ml 70% ethanol. After the initial fixation, cells were suspended in 0.5 ml PBS containing 25 μg/ml propidium iodide (PI), 0.2% Triton X-100 and 40 μg/ml RNase A and incubated for at least 30 minutes at 4°C. Cells were then analyzed for DNA content profile by flow cytometry (FACScan, Becton Dickinson, San Jose, CA) from 10,000 events per sample. Data from flow cytometry were analyzed using WinList software (Verity Software House Inc., Topsham, ME) and presented as DNA content profiles (X axle) over cell numbers (y axle). Triplicate assays were performed.

### Western blot analysis

Cells with and without bortezomib treatment were washed with phosphate-buffered saline (PBS) and lysed on ice for 30 minutes in PBS containing 1% Nonidet P-40, 0.5% sodium deoxycholate, 0.1% sodium dodecyl sulfate (SDS), 10 μg/ml phenylmethyl sulfonyl fluoride, and 20 μM leupeptin. Cell lysates were then centrifuged at 15,000 g for 20 minutes at 4°C. Fifty μg total proteins from each sample were heated at 95°C for 5 minutes after mixing with equal volume of 2 × SDS loading buffer. Samples were separated on 12 - 15% SDS-polyacrylamide gel electrophoresis (SDS-PAGE) gels and electrotransferred to Pure Nitrocellulose Membranes (Bio-Rad, Hercules, CA). The membrane was then blocked in 5% skim milk in TBS-T buffer (20 mM Tris/HCl (pH 7.5), 0.137 M NaCl, and 0.05% Tween 20) at room temperature for 2-3 hours; followed by incubation of the membrane with primary antibodies (against survivin or actin) in TBS-T containing 5% BSA overnight at 4°C in the range of dilutions from 1:1000 to 1:4000. After washing with TBS-T, the membrane was incubated in TBS-T buffer containing 5% skim milk containing the corresponding secondary antibody (1:5000) for 45-60 minutes at room temperature with shaking. Protein of interest was detected using ECL (Perkin Elmer, Waltham, MA) and visualized by autoradiography with various times (5-60 seconds) of exposure. Actin was detected as the internal control for normalization of total protein loading in each lane.

### Cell death detection ELISA assay

This assay is based on cell DNA fragmentation and the cell death/DNA fragmentation was detected using the Cell Death Detection ELISA^Plus ^assay kit (Roche) as described previously [37]. Briefly, transfected HCT116p53-/- cells were seeded in triplicates in 96-well plates and treated with and without bortezomib for 48 hours. After removing medium, cells were then lysed and 20 μl of lysate supernatant from each well were dispensed into streptavidin-coated well-removable 96-well plates followed by addition of 80 μl of immuno-reagents. After a 2-hour incubation at room temperature, unbound components were removed by washing with 1× incubation buffer for 3 times, followed by adding 100 μl of HRP substrate to each well, and the plate was placed on a shaker at 250 rpm for color development. Measurements were made at 405 nm against an ABTS solution as a blank control using a microplate reader. The absorbance value at 405 nm represents the quantities of DNA fragments/apoptosis induced by the treatment.

### Statistical analysis

A t-test was performed for a pair-wise comparison of each experimental pair group with the control assuming equal variance. The significance (p-value marked with an asterisk "*") was set at equal to or less than 0.05.

## Results

### Bortezomib-induced growth inhibition in HCT116 colon cancer cells is dependent on p53 statuses

**Figure 1:**
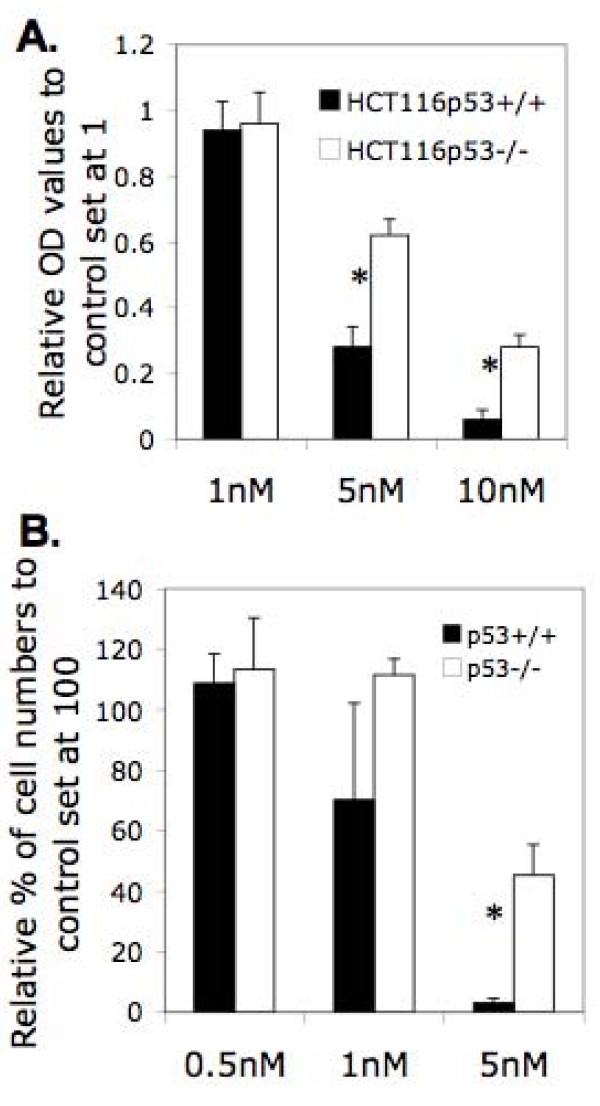
**Colon cancer cell growth inhibition by bortezomib**. HCT116 colon cancer cells with p53 wild type (p53^+/+^) and p53 null (HCT116p53^-/-^) were treated with bortezomib at different concentrations for 48 hours. Cell growth was determined by MTT assay (**A**) or by direct cell counting (**B**). The resultant data were plotted in histogram by setting no bortezomib treatment controls as OD values at 1 (A) or as cell numbers at 100. Each bar represents the mean ± SD derived from three independent determinations.

It was found that wild type p53 transcriptionally inhibits survivin expression [27-29]. We reasoned that if survivin plays a role in bortezomib resistance, p53 status might affect bortezomib sensitivity to inhibit cancer cell growth. Consistent with our rationale, p53 null HCT116 cells (HCT116p53^-/-^) were resistant to bortezomib-induced cell growth inhibition in comparison with HCT116 with wild type p53 (Fig 1). This suggests a role for the p53 status in bortezomib-induced cancer cell growth inhibition, however it is not known whether the difference of p53 status can also affect bortezomib-induced cell death.

### HCT116p53^-/- ^colon cancer cells are much more resistant to bortezomib-mediated cell death in comparison with wild type HCT116 cells

**Figure 2:**
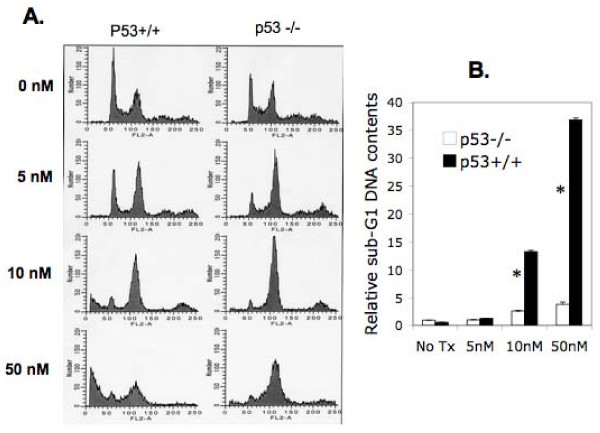
**Colon cancer cell death induced by bortezomib**. Sub-confluent HCT116 and HCT116p53^-/- ^cells were treated with and without bortezomib at different concentrations as shown for 24 hours. Cells were then collected and stained with PI, followed by flow cytometry analysis of DNA content profiles. **A**. The flow cytometry resultant data in histogram showed the striking difference in DNA content profiles between HCT116 cells and HCT116p53^-/- ^cells. **B**. Histogram to compare the different percentage of cells in sub-G1 (dead cells) between HCT116 cells and HCT116p53^-/- ^cells. The histogram in B is the mean ± SD derived from two independent determinations.

We then tested the effect of bortezomib on the induction of HCT116 colon cancer cell death with different p53 status. Flow cytometry was used to determine DNA content profiles as a parameter to evaluate cell death after bortezomib treatment. This experiment revealed that bortezomib treatment for 24 hours at 10 and 50 nM significantly induced sub-G1 DNA (representing dead cells) content increase in HCT116p53^+/+ ^cells, while this treatment showed minimal effect on HCT116p53^-/- ^cells (Fig. 2). These observations (Figs 1 and 2) prompted us to investigate the potential role for survivin in bortezomib resistance.

### Survivin expression is much higher in HCT116p53^-/- ^cells than in HCT116p53^+/+ ^cells

**Figure 3:**
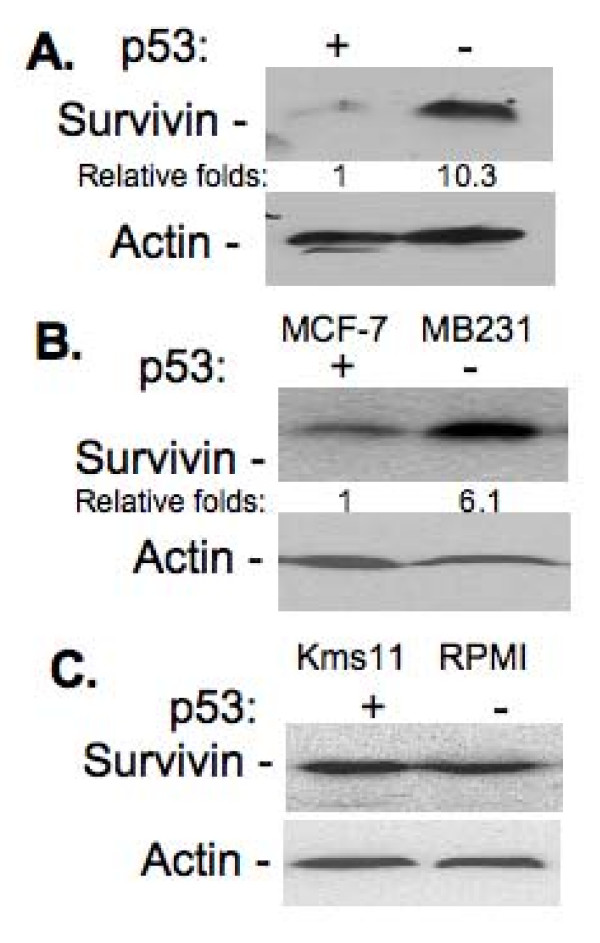
**Survivin Expression in wild type vs. p53 null cancer cell sublines**. **A**. HCT116 and HCT116p53-/-. **B**. MCF-7 with wild type p53 and MDA-MB-231 with mutant p53. **C**. Kms11 with wild type p53 and RPMI-8226 with mutant p53. Sub-confluent cells were lysed, and the cell lysates were used to determine survivin expression by western blots. Actin is the internal control for total protein loading. The expression of survivin in wild type p53 cells was set at 1 and relative survivin expression is shown after normalization with the actin internal control.

We reasoned that if survivin plays a role in bortezomib resistance, survivin expression would be lower in HCT116p53^+/+ ^cells than in HCT116p53-/- cells. Alternatively, bortezomib may decrease survivin expression in HCT116p53^+/+ ^cells or increase survivin expression in HCT116p53^-/- ^cells. To test the former possibility, survivin expression was determined by western blots in both HCT116p53^+/+ ^cells and HCT116p53^-/- ^cells. The result indicated that the expression of survivin in HCT116p53^+/+ ^cells is much lower than in HCT116p53^-/- ^cells (Fig. 3A), suggesting the high expression of survivin in HCT116p53^-/- ^cells may act as a contributing factor to bortezomib resistance. Similar results were obtained in other cancer cell lines with different p53 status (Fig. 3B). Consistently, MDA-MB-231 has much higher tumorigenic ability than MCF-7 in mouse xenograft models.

### Bortezomib induces survivin expression in HCT116p53^-/- ^cells but shows no significant effect on survivin expression in HCT116p53^+/+ ^cells

**Figure 4:**
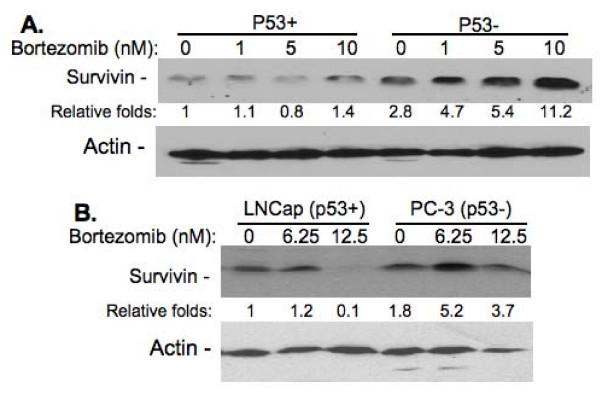
**Differential effects of bortezomib on survivin in HCT116p53^-/- ^cells versus HCT116 cells**. **A**. HCT116 and HCT116p53-/-. **B**. LNCap with wild type p53 and PC-3 with null p53. Sub-confluent cells were treated with and without bortezomib for 48 hours. Cells were then collected and lysed for western blots to determine survivin expression. Actin was used as the internal control for total lysate protein loading. The expression of survivin in wild type p53 cells was set at 1 and relative survivin expression is shown after normalization with actin.

We then tested whether bortezomib could differentially modulate survivin expression between HCT116p53^+/+ ^cells and HCT116p53^-/- ^cells. Consistent with the fact that HCT116p53^-/- ^cells are resistant to bortezomib-induced growth inhibition and apoptosis induction, bortezomib appears to significantly induce survivin expression in HCT116p53^-/- ^cells, while it shows minimal induction of survivin in HCT116p53^+/+ ^cells (Fig. 4A). Similar results were also obtained in other cancer cell lines (Fig. 4B), indicating a general principle of this phenomenon.

### Silencing of survivin expression in HCT116p53^-/- ^cells by survivin mRNA-specific siRNA sensitizes bortezomib-induced growth inhibition

**Figure 5:**
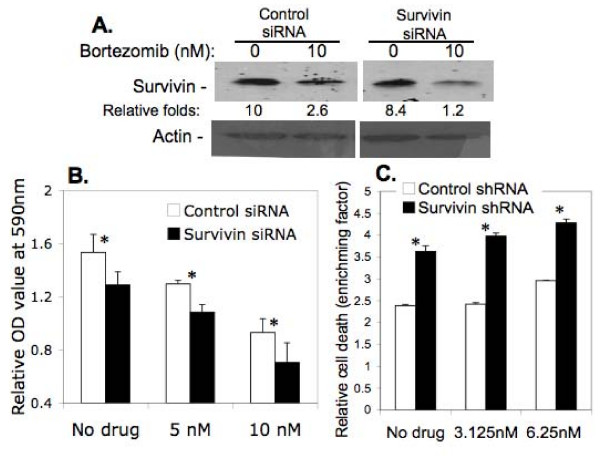
**Effects of silencing of survivin expression on bortezomib sensitivity in HCT116p53-/- cells**. The highly survivin expressing HCT116p53-/- cells at 50% confluence were transfected with survivin mRNA-specific siRNAs or with control siRNAs. After 16 hours post transfection, cells were treated with and without bortezomib for 48 hours. A part of the transfected cells were then collected for western blots to determine survivin expression (**A**), a part of the transfected cells was used to determine cell growth inhibition by MTT assay (**B**), and the other part of the transfected cells was used to determine cell death/DNA fragmentation by cell death ELISA assay (**C**). Data shown in B and C are the mean ± SD derived from three independent determinations. Note: Results from cells without transfection were similar to cells transfected with control siRNA/shRNA (not shown). The expression of survivin in HCT116p53-/- cells was set at 10 and relative survivin expression levels are shown after normalized to actin.

**Figure 6:**
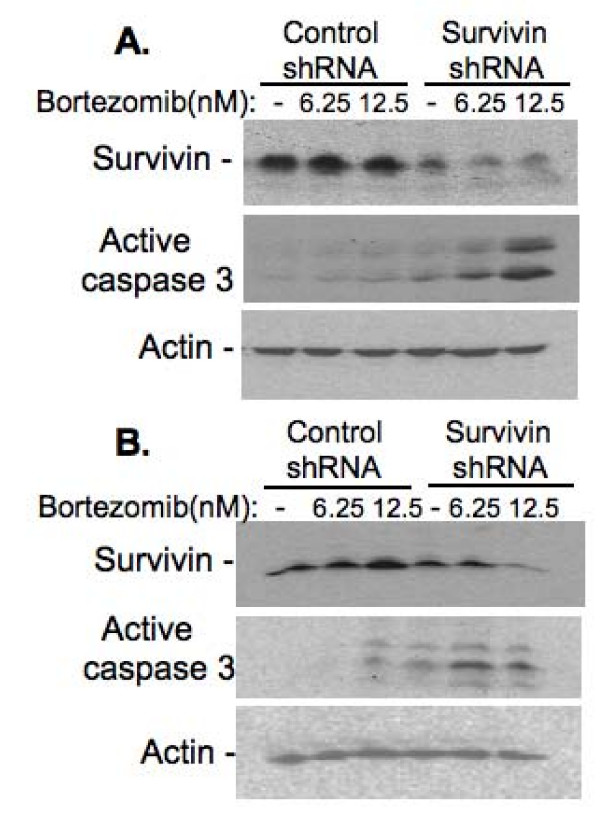
**Effects of silencing of survivin expression on bortezomib sensitivity in other cancer cell with mutant p53**. Cell treatment condition is the same as in Figure 5. Cells were then collected for western blots to determine survivin expression and/or caspase-3 activation. **A**, MDA-MB-231 breast cancer cells are with mutant p53. **B**, PC-3 prostate cancer cells are with p53 null.

To test whether survivin expression indeed plays a role in bortezomib resistance, we employed survivin mRNA-specific siRNA approach [35] to silence survivin expression in HCT116p53^-/- ^cells, which highly expresses survivin. Significantly, we noted that silencing of the expression of survivin (Fig. 5A) reverses bortezomib resistance to growth inhibition (Fig. 5B) and cell death induction (Fig. 5C) in HCT116p53-/- cells, while control siRNA only showed a background effects similar to those without transfection (not shown). Similarly, silencing of survivin expression in MDA-MB-231 (p53 mut) and PC-3 (p53 null) cells activates caspase-3 (Fig. 6), a hallmark of apoptosis. These studies provide direct evidence for the involvement of survivin expression in bortezomib resistance.

### Cancer cell sensitivity to bortezomib treatment is dependent on p53 status but not cancer cell types

**Figure 7:**
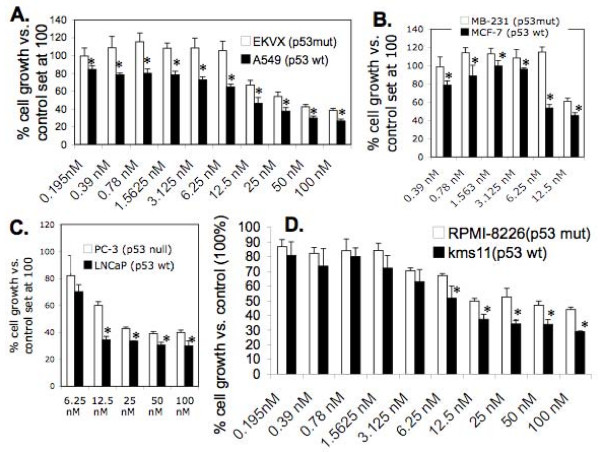
**p53 status but not cancer cell type is a critical indicator for bortezomib sensitivity**. Lung cancer cell lines (**A**, EKVX with mutant p53 and A549 with wild type p53), breast cancer cell lines (**B**, MDA-MB-231 with mutant p53 and MCF-7 with wild type p53), prostate cancer cell lines (**C**, PC-3 with null p53 and LNCaP with wild type p53) and myeloma cancer cell lines (**D**, RPMI-8226 with mutant p53 and KMS11 with wild type p53) were seeded in 96-well cell cultural plates at 30% confluence. Cells were treated with and without a series of bortezomib concentrations for 48 hours 16 hours after seeding. Cell growth/survival was then determined by MTT assay. The resultant data were represented in histograms. Each bar is the mean ± SD derived from three independent determinations.

Previous studies indicated that modulation of survivin expression by bortezomib, and cancer cell sensitivity to bortezomib-induced apoptosis are cell type-dependent [34]. Based on the data provided above, we hypothesized that the different sensitivity to bortezomib for cancer cells is due to p53 status-associated differential survivin expression, and induction by bortezomib, rather than cancer cell type. Here, we tested four pairs of cancer cell lines with different p53 status from lung cancer (EKVX with mutant p53 versus A549 with wild type p53), breast cancer (MDA-MB-231 with mutant p53 versus MCF-7 with wild type p53), prostate cancer (PC-3 with null p53 versus LNCaP with wild type p53) and myeloma (RPMI-8226 with mutant p53 versus Kms11 with wild type p53). Consistent with our early data and our rationale, bortezomib-mediated inhibition of cell growth is significantly better in cancer cell lines with wild type p53 in comparison to those cell lines with a p53 null or p53 mutant status (Fig. 7), which is consistent with the relative expression level of survivin in these cells (Fig. 3A and 3B).

## Discussion

Bortezomib is the first in class, proteasome inhibitor that has demonstrated significant anticancer activity in patients with lymphoid malignancies especially multiple myeloma [38,39]. However, growing studies indicated the potential effectiveness of bortezomib in treatment of patients with solid tumor including colon-gastric cancer [1-3], breast cancer [4-9], prostate cancer [10-14] and lung cancer [15-18]. However, despite its impressive single agent clinical activity in patients with either hematopoietic or solid malignancy, most patients either fail to respond or develop resistance to bortezomib treatment. Therefore, resistance to bortezomib is a challenging problem in the clinic. Identifying mechanism of bortezomib resistance not only can help identify novel therapeutic targets but will also contribute to better utilization of this important therapeutic agent.

In the present study, we focus on the role of survivin and p53 in bortezomib effectiveness as well as their functional relationship in solid tumor cell lines. We found that cancer cells with wild type p53 express much less survivin in comparison with cancer cells with either mutant or null p53. Moreover, bortezomib significantly increased survivin expression in the HCT116 colon or other cancer cell lines with p53 null, while it only showed a minimal effect on survivin expression in HCT116 and other cancer cells with wild type p53. Consistent with these findings, while bortezomib effectively inhibited cell growth and induced cell death in cancer cells with wild type p53, bortezomib showed ineffectiveness to inhibit cell growth and induce cell death for the cancer cells with abnormal p53 (null or mutated). We recognized that our experiment in Fig. 7 will be more convincing, if pairs of cancer cell lines as we have for the HCT116 line (HCT116p53^+/+ ^vs. HCT116p53^-/-^) could be available to us for these experiment. Nevertheless, the role of survivin in bortezomib resistance was directly demonstrated in the study by silencing of survivin in several cancer cell lines with mutant p53 using survivin mRNA-specific siRNA/shRNA technology previously set up in our laboratory [35,36]. Finally, our investigations in three different pair of cancer cell lines (originating from breast, lung and prostate) with different p53 status demonstrated that the p53 status-associated survivin expression is an essential parameter to predict bortezomib resistance irrespective of the origin of the cancer cell. Cancer cells having a wild type p53 were sensitive while those with abnormal p53 (mutated or null) were resistant to bortezomib treatment. Consistent with these findings, previous studies found that wild type p53 transcriptionally inhibits survivin expression in various cancer cell types [27-29] and bortezomib can stabilize wild type p53 in prostate cancer cells [40]. Here, we would like to point out that the bortezomib concentration used affects the results, suggesting the dose used in the clinic should be carefully considered. When high dose may kill cancer cells better in a short term, high dose will increase the possibility to generate bortezomib resistance, suggesting that in addition to p53 status-associated survivin expression, other factors, such as other protein members in the IAP and Bcl-2 families may also play important roles in bortezomib resistance. Nevertheless, we have confirmed a role for survivin in bortezomib resistance by direct silencing of survivin expression using survivin-specific siRNA/shRNA. This finding is significant because our recent studies indicated that survivin may be a superior cancer stem cell marker and possibly plays critical role in cancer stem cell expansion [41]. In this regard, cancer cells appear to have a higher percentage of subpopulation cells that are tumorigenic (cancer initiating/cancer stem cells) in xenograft mouse models [42].

Therefore, consideration of both survivin expression and p53 status as interconnecting biomarkers and targets in cancer cells may not only be useful for predicting the outcome of bortezomib treatment, but may also provide pivotal criteria for rational drug combination. For example, bortezomib likely induces survivin expression in cancer cells with mutated or null p53 (this study), and it is known that paclitaxel rapidly induces survivin expression [43]. Thus, combination of bortezomib and paclitaxel likely obtained no good results in many cancer types with such as the mutated p53 background. Accordingly, a recent Phase II study in patients with metastatic esophageal, gastric, and gastroesophageal cancer showed poor results in the drug combination [44]. However, it is also possible that the poor results derived from such a drug combination involve other mechanisms of drug resistance in these tumors that are notoriously difficult to treat with chemotherapy.

An important question that needs be answered for better application of the findings is the mechanism underlying bortezomib-mediated induction of survivin expression in mutated or null p53 cancer cells, while it showed downregulation of or minimal effect on survivin expression in wild type p53 cancer cells. Although answering this critical question will need further research efforts, based on the current available information, the potential p53 and NF-κB functional crosstalk could provide a plausible explanation, although need to be further confirmed. As reviewed before, the survivin gene is a potential downstream target for p53 and NF-κB transcriptional regulation [26]. Alternatively, the previous finding that bortezomib stabilizes active form of p53 in human LNCaP-Pro5 prostate cancer cells may provide another explanation [40]. Nevertheless, while survivin expression is inhibited by wild type p53 [27-29], survivin and NF-κB appear to be co-expressed in cancer such as in peripheral T-cell lymphoma [45], and inhibition of NF-κB activity using NF-κB-specific inhibitors decreased survivin expression [46]. Consistent with these observations, bortezomib resistance requires NF-κB activity in mantle cell lymphoma [47]. Therefore, the potential connection of these factors provide an interesting underlying mechanism, which is likely similar to the mechanism we recently discovered for the p53 and ERα on the survivin gene control in the breast cancer [30].

Finally, the p53 status in RPMI-8226 and Kms11 is not fully consistent in literature. Our literature search indicates that RPMI-8226 has mutant p53 [48], while Kms11 has wild type p53[49]. However, some publication indicated that Kms11 is p53 null. This is likely due to the hypermethylation of the p53 gene to make p53 expression extremely low [50]. Consistently, our results (Li and Chanan-Khan, unpublished observation) indicated that the expression of p53 in Kms11 was barely detected. Consistent with this, we found that the expression of survivin in Kms11 is comparable with its level in RPMI-8226 (Fig. 3C).

## Conclusion

In conclusion, based on the finding in this study, survivin appears to play a role in bortezomib resistance. The p53 status-associated survivin expression is an important parameter for predicting bortezomib sensitivity, which is largely independent of cancer cell types. Therefore, the finding in this paper should be useful for not only prediction of bortezomib sensitivity, but may also be useful as an essential criterion for bortezomib combination with other anticancer compounds for treatment of cancer patients.

## Abbreviations

bortezomib: PS-341 OR velcade^®^; IAP: inhibitor of apoptosis; MTT: tetrazolium salt, 3-[4,5-dimethylthiazol-2-yl]-2,5,-diphenyltetrazolium bromide; NSCLC: non-small cell lung cancer; siRNA: small interference RNA.

## Competing interests

The authors declare that they have no competing interests.

## Authors' contributions

XL carried out the experimental design, performed most of the experiments and organized data for manuscript. DC performed the rest of experiments and involved in results discussion and organization. AAC initiated bortezomib-related projects in our institute, helped experimental design and revised the manuscript. FL initiated the project, participated in experimental design and wrote the manuscript. All authors read and approved the final manuscript.

## Author information

Diane Calinski was a student in the Roswell Park Summer College Student Program at the time for this work.
